# Mendelian randomization analysis identifies druggable genes and drugs repurposing for chronic obstructive pulmonary disease

**DOI:** 10.3389/fcimb.2024.1386506

**Published:** 2024-04-10

**Authors:** Zihui Wang, Shaoqiang Li, Guannan Cai, Yuan Gao, Huajing Yang, Yun Li, Juncheng Liang, Shiyu Zhang, Jieying Hu, Jinping Zheng

**Affiliations:** National Center for Respiratory Medicine, National Clinical Research Center for Respiratory Disease, Guangzhou Institute of Respiratory Health, the First Affiliated Hospital of Guangzhou Medical University, Guangzhou Medical University, Guangzhou, China

**Keywords:** Mendelian randomization, druggable genes, drugs repurposing, chronic obstructive pulmonary disease, lung function

## Abstract

**Background:**

Chronic obstructive pulmonary disease (COPD) is a prevalent condition that significantly impacts public health. Unfortunately, there are few effective treatment options available. Mendelian randomization (MR) has been utilized to repurpose existing drugs and identify new therapeutic targets. The objective of this study is to identify novel therapeutic targets for COPD.

**Methods:**

Cis-expression quantitative trait loci (cis-eQTL) were extracted for 4,317 identified druggable genes from genomics and proteomics data of whole blood (eQTLGen) and lung tissue (GTEx Consortium). Genome-wide association studies (GWAS) data for doctor-diagnosed COPD, spirometry-defined COPD (Forced Expiratory Volume in one second [FEV1]/Forced Vital Capacity [FVC] <0.7), and FEV1 were obtained from the cohort of FinnGen, UK Biobank and SpiroMeta consortium. We employed Summary-data-based Mendelian Randomization (SMR), HEIDI test, and colocalization analysis to assess the causal effects of druggable gene expression on COPD and lung function. The reliability of these druggable genes was confirmed by eQTL two-sample MR and protein quantitative trait loci (pQTL) SMR, respectively. The potential effects of druggable genes were assessed through the phenome-wide association study (PheWAS). Information on drug repurposing for COPD was collected from multiple databases.

**Results:**

A total of 31 potential druggable genes associated with doctor-diagnosed COPD, spirometry-defined COPD, and FEV1 were identified through SMR, HEIDI test, and colocalization analysis. Among them, 22 genes (e.g., MMP15, PSMA4, ERBB3, and LMCD1) were further confirmed by eQTL two-sample MR and protein SMR analyses. Gene-level PheWAS revealed that ERBB3 expression might reduce inflammation, while GP9 and MRC2 were associated with other traits. The drugs Montelukast (targeting the MMP15 gene) and MARIZOMIB (targeting the PSMA4 gene) may reduce the risk of spirometry-defined COPD. Additionally, an existing small molecule inhibitor of the APH1A gene has the potential to increase FEV_1_.

**Conclusions:**

Our findings identified 22 potential drug targets for COPD and lung function. Prioritizing clinical trials that target these identified druggable genes with existing drugs or novel medications will be beneficial for the development of COPD treatments.

## Introduction

Chronic Obstructive Pulmonary Disease (COPD) is a complex lung condition characterized by persistent progressive airflow limitation, along with chronic respiratory symptoms (dyspnea, cough, sputum, etc.). Recent large-scale studies have revealed that the global prevalence of COPD among people aged 30-79 years was estimated to be 10.3% in 2019, with a staggering 391.9 million patients worldwide (Adeloye et al., 2022). The prevalence is expected to continue rising due to factors such as increasing smoking rates, air pollution, and an aging population. In addition, COPD ranks as the third leading cause of death globally, with approximately 2.5 deaths per minute worldwide, and a large proportion of deaths are caused by infections (S. European Observatory on Health, Policies et al., 2023). Given the high burden of COPD-related mortality and morbidity, it is necessary to implement interventions to reduce its prevalence and minimize the associated disease burden.

Despite extensive research, the treatment options for COPD remain limited. Currently, the main treatment involves bronchodilators, hormones, and mucolytic agents. However, these drugs only provide temporary relief of symptoms and modest improvements in lung function without significantly impacting the overall prevalence of the disease. Moreover, existing drugs are ineffective in controlling the symptoms and disease progression in some patients. Therefore, there is an urgent need to develop new therapeutic drugs.

**Figure 1:**
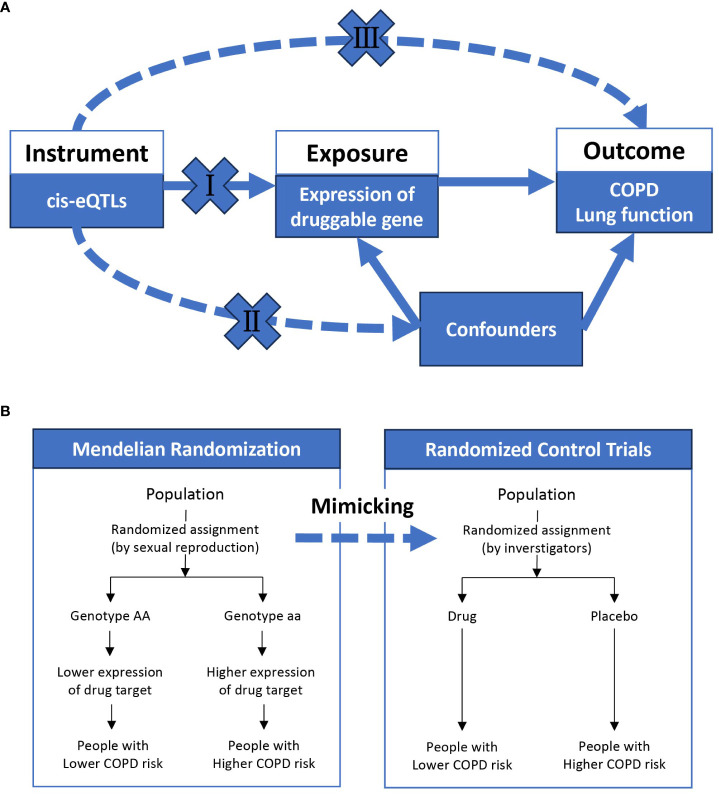
The overview of Mendelian randomization. **(A)** shows the basic assumptions underlying Mendelian randomization: (I) genetic variation must be associated with exposure; (II) genetic variation must not be associated with any confounding factors; (III) genetic variation must not be directly related to outcome. **(B)** indicates that Mendelian randomization is similar to a randomized controlled trial, where different genotypes (on autosomes) are randomly assigned from one generation to the next according to Mendel’s law, and a population randomly assigned to a genotype with high expression of a particular gene will be exposed to high expression of a particular gene for life. COPD, chronic obstructive pulmonary disease; eQTL, expression quantitative trait loci.

Incorporating genetics into drug development shows promise in addressing this issue, as genetically-supported drugs are more likely to be successful in clinical trials (Trajanoska et al., 2023). For instance, the identification of the PCSK9 gene’s association with coronary artery disease risk has led to the development and successful application of PCSK9 inhibitors (Stoekenbroek et al., 2015). Druggable genes refer to those that can be targeted by drugs, including small molecules and biotherapeutic agents, to modulate their activity (Finan et al., 2017). Genome-wide association studies (GWAS) have been effective in identifying single nucleotide polymorphisms (SNPs) associated with COPD (Silverman, 2020). However, GWAS may fall short in pinpointing causative genes for direct drug development. In this context, Mendelian randomization (MR) provides a more reliable approach by mimicking the effects of gene overexpression, similar to randomized controlled trials (**Figure 1**). As a result, MR informs drug development strategies (Schmidt et al., 2020) and has been employed to explore potential opportunities for drug repurposing (Folkersen et al., 2020; Gaziano et al., 2021; Ou et al., 2021; Storm et al., 2021).

In this study, we hypothesized that increased expression of the druggable gene in whole blood (exposure) could modify the risk of developing COPD (outcome). We extracted cis-expression quantitative trait loci (cis-eQTL) and protein quantitative trait loci (pQTL) for 4,317 druggable genes to investigate the causal effects of druggable gene expression on doctor-diagnosed COPD, spirometry-defined COPD (Forced Expiratory Volume in one second [FEV1]/Forced Vital Capacity [FVC]<0.7), and FEV1. Furthermore, the safety of the identified potential druggable genes and opportunities for repurposing licensed or clinical-stage drugs in COPD were assessed.

## Methods

### Overall design

**Figure 2:**
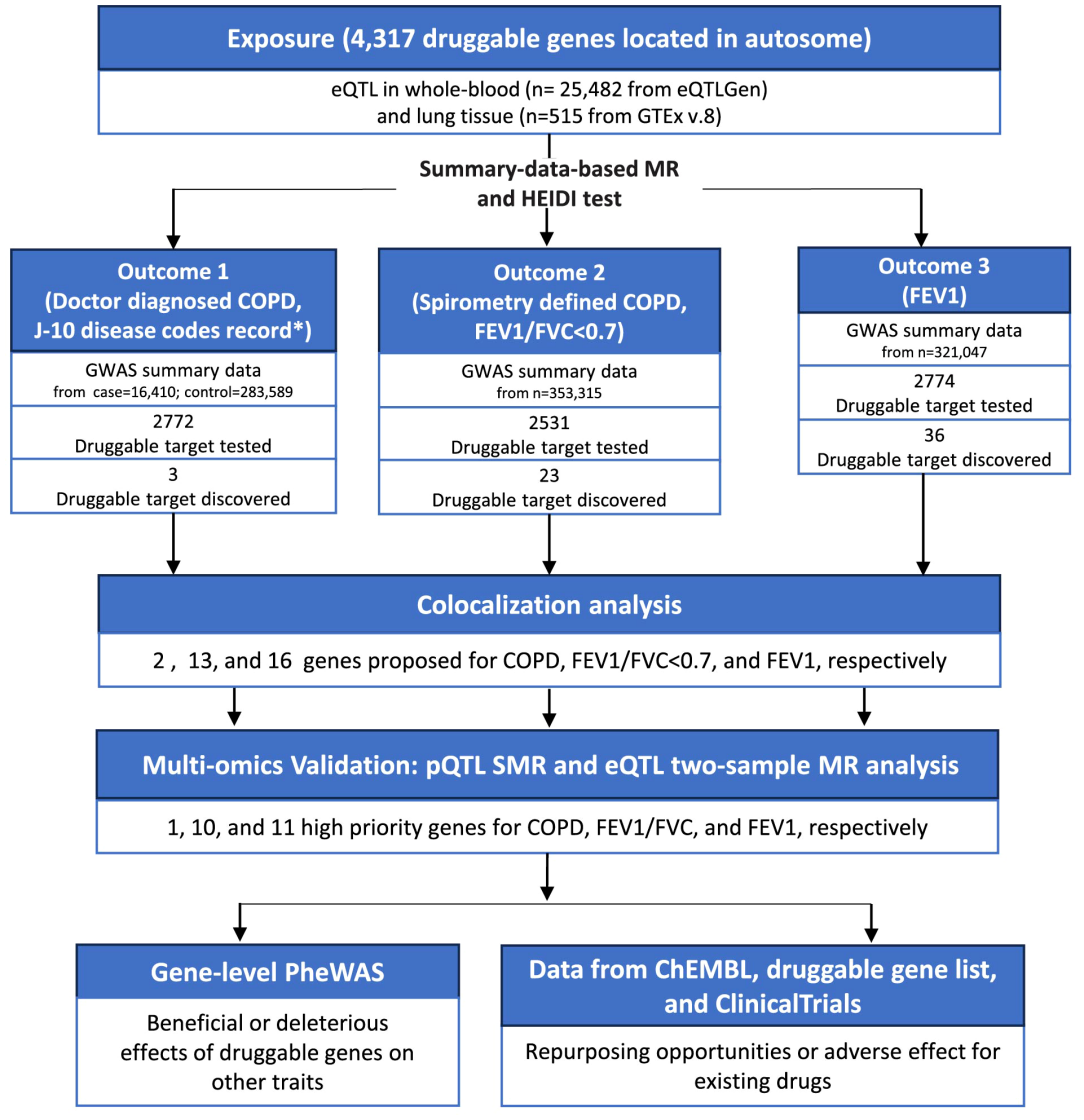
The flowchart of this study. COPD, chronic obstructive pulmonary disease; eQTL, expression quantitative trait loci; MR, Mendelian randomization; GWAS, genome-wide association studies; FEV1, forced expiratory volume in 1 second; FVC, forced vital capacity; pQTL, protein quantitative trait loci; SMR, summary-data-based mendelian randomization; HEIDI, heterogeneity in dependent instruments; PheWAS, phenome-wide association studies.

We observed the hospitalization of 56 patients with COPD to observe the real-life disease burden of COPD on patients and to demonstrate the importance of drug development. The overall scheme of COPD drug target screening was shown in **Figure 2**. Briefly, we conducted a Summary-data-based Mendelian Randomization (SMR) analysis, using genetic variants that affect the expression of druggable genes in blood and lung tissue as instrumental variables to estimate the causal relationships between these genetic variations and doctor-diagnosed COPD, FEV1/FVC<0.7, and FEV1. The SMR method utilizes summary-level data from GWAS and QTL studies to test for associations between gene expression levels and complex traits of interest. Two-sample MR estimates the causal effects by separately estimating the associations between instrumental variables and both exposure factors and outcomes, requiring two sets of independent sample data. SMR can be used to prioritize potential genes for further research, while two-sample MR is suitable for causal inference. For SMR results meeting the significance threshold and passing the heterogeneity independent instrument (HEIDI) test, we conducted colocalization analyses to determine if the same SNPs were driving both the outcome and exposure. Furthermore, eQTL two-sample MR and protein SMR analyses were employed to ensure consistency in the results. Finally, we matched existing drugs to the identified druggable genes and conducted a PheWAS analysis to preliminarily evaluate the safety of these genes as potential drug targets (i.e., whether they may cause other diseases).

This study used the big data platform of the Clinical Medical Research Centre of the First Affiliated Hospital of Guangzhou Medical University, and the patient’s clinical data were obliterated for privacy-related information before they were provided to the investigators. The observational study was approved by the Ethics Committee of the First Affiliated Hospital of Guangzhou Medical University (ES-2023-017-01).

### Druggable genes list

Druggable genes were obtained from a review by Finan et al. (Finan et al., 2017). A total of 4317 druggable genes on autosomes were utilized in this study.

### Exposure data

The eQTL data for whole blood were obtained from the eQTLGen consortia (www.eQTLgen.org), while the lung-specific eQTL data were obtained from the GTEx v.8 consortia (www.gtexportal.org). Detailed descriptions of the data can be found in the original publications (Aguet et al., 2020; Võsa et al., 2021). The eQTLGen consisted of a total of 25,482 whole blood samples from 37 datasets, with the majority of samples being of European ancestry. Gene expression levels of the samples were analyzed by Illumina, Affymetrix expression arrays, and RNA-seq. The GTEx version 8 data contains lung tissue samples from 515 postmortem donors, the majority of whom were of European ethnicity. The mRNA from each tissue sample was sequenced at a median depth of 82.6 million reads. We Used eQTLGen and GTEx v.8 to identify all conditionally independent eQTLs in whole blood and lung tissue, which covered 2531 of the 4317 druggable genes in at least one tissue. The pQTL data for whole blood were obtained from Ferkingstad et al. study, which contained pQTLs for 4907 plasma proteins (Ferkingstad et al., 2021).

For the SMR analysis, we downloaded the cis-eQTL results from eQTLGen and GTEx v.8 consortia in SMR format in May 2023 (yanglab.westlake.edu.cn/software/smr). In the latest version, every SNP-gene combination with a distance <1Mb was included. To further validate the effect of druggable genes at the protein level, we extracted available cis-pQTLs from the study by Ferkingstad et al., considering those with a significance threshold of *P* < 5e−8.

For the two-sample MR analysis, the cis-eQTLs, which were defined as significant (5e-8) SNPs located within 5 kb upstream of the starting point or 5 kb downstream of the endpoint of a druggable gene, were extracted from the eQTLGen and GTEx v.8 consortia.

### Outcome data

FEV1 quantifies the maximal volume of air expelled in the first second of a forceful exhalation, serving as a critical parameter for evaluating pulmonary function. A reduced FEV1/FVC ratio (<0.7) typically indicates airflow obstruction (spirometry defined COPD). The doctor diagnosed COPD in this study comes from the J-10 disease codes record of the healthcare system. This diagnosis incorporates an assessment based on the patient’s exposure history (such as smoking), symptoms (coughing, expectoration, or dyspnea), and post-bronchodilator pulmonary function tests. Conversely, spirometry-defined COPD (with a pre-bronchodilator FEV1/FVC<0.7) is identified during population health screenings. Participants meeting these conditions may not have any exposure history or symptoms (accounting for more than 70% of cases) (Fang et al., 2018) and did not undergo post-bronchodilator pulmonary function tests, making them a distinctly different cohort from those diagnosed with COPD by a doctor. Consequently, this study separately investigates the druggable genes associated with both doctor-diagnosed COPD and spirometry-defined COPD (pre-bronchodilator FEV1/FVC<0.7). All doctor-diagnosed COPD data were obtained from the FinnGen consortium (version R8, downloaded in May 2023), which comprises 16,410 patients with COPD and 283,589 controls (differentiated according to J-10 disease codes in the medical electronic system). The cohorts for FEV1 and FEV1/FVC<0.7 were derived from the UK Biobank and SpiroMeta consortium. For further details regarding the data, please refer to the original literature and the official website (**Supplementary Table 1**) (Shrine et al., 2019; Higbee et al., 2021).

### Summary-data-based Mendelian randomization

The MR analysis is based on three fundamental assumptions: 1) genetic variation must be associated with the exposure; 2) genetic variation must not be associated with any confounding factors; and 3) genetic variation must not have a direct relationship with the outcome (Sanderson et al., 2022). As an extension of the MR concept, SMR employs two-step least squares (2SLS) to estimate the effect of the exposure on the outcome (e.g., doctor diagnosed COPD) using genetic variation that is significantly associated with the exposure (e.g., genes targetable for drug intervention at the transcriptional or protein expression level) (Sanderson et al., 2022). The causal associations were computed as follows:


$$\beta_{druggable\;gene-COPD}=\;\beta_{SNP-COPD}/\;\beta_{SNP-druggable\;gene}$$

The odds ratio (OR) estimates of the effect of druggable gene expression on COPD risk are calculated using the formula: OR_druggable gene-COPD_=exp(β_druggable gene-COPD_), where OR represents the odds ratio estimate per 1-ln increase in the level of the druggable gene, and exp is the base of the natural logarithm.

SMR analyses were completed using the SMR software for Linux version 1.0.3 with default options (https://yanglab.westlake.edu.cn/software/smr). To account for multiple testing and control for genome-wide type I error rates, we used the Bonferroni correction to adjust the *P* values (Armstrong, 2014).

### HEIDI test

We conducted the HEIDI test to assess whether the observed associations were influenced by vertical pleiotropy rather than linkage disequilibrium (LD) of the dependent variable (Zhu et al., 2016). LD estimation was performed using the European pedigree genomes from the 1000 Genomes Project Consortium as a reference (Zheng-Bradley and Flicek, 2017). Associations with a significance level of *P* < 0.01 in the HEIDI test were excluded as they may be attributed to LD rather than pleiotropy.

### Colocalization

An alternative Bayesian test was performed to examine the colocalization of the two traits and estimate the posterior probability of shared variation (Giambartolomei et al., 2014). For each leading SNP in the outcome GWAS database, we retrieved all SNPs within a 100 kb range upstream and downstream. The posterior probability of H4 (PP.H4) was estimated, with the commonly used threshold of PP.H4 > 0.8 for GWAS and QTL associations (Navarro et al., 2021).

### Two-sample Mendelian randomization

To further validate the results of the SMR analysis, the two-sample MR approach was employed to estimate the effect on outcomes by using eQTL of druggable genes as instruments (Li et al., 2023). We assessed the strength of the correlation between instrumental variables and exposure using the F-statistic (>10) (Burgess and Thompson, 2011). In the presence of directional pleiotropy, we utilized the MR-egger estimated effect size as the outcome (Burgess and Thompson, 2017). The study proceeded with the two-sample MR analysis using MR-Egger, weighted median, inverse variance weighting, simple mode, and weighted mode methods. When multiple analytical methods yield similar results, we consider the MR results to be more robust. Cochran’s Q test was used to assess the overall heterogeneity of Wald ratios (Higgins et al., 2003). In addition, the Steiger test was conducted to determine whether the exposure precedes the outcome (https://jean997.github.io/cause) (Hemani et al., 2017).

### Phenome-wide Mendelian randomization

To further assess the potential horizontal pleiotropy and any possible side effects of potential drug targets, we conducted a PheWAS analysis using the AstraZeneca portal (https://azphewas.com/). The original study utilized data from approximately 15,500 binary phenotypes and around 1,500 continuous phenotypes from about 450,000 participants who underwent exome sequencing, as published by the UK Biobank. The complete methodology can be found in the original article (Wang et al., 2021). We performed multiple corrections and set the threshold at 2E-9 to account for possible false positives.

All DNA positions are based on the human reference genome build hg19 (GRCh37). Data processing was carried out using R software version 4.2 with “TwosampeMR” and “easyMR” packages.

### Metagenomic next-generation sequencing (mNGS) pipeline

mNGS and hybridization capture-based targeted mNGS were used to detect pathogens. Briefly, about 1 mL of sample was centrifuged at 12,000 g for 5 min to collect the pathogens and human cells. Next, 50 μL of precipitate underwent depletion of host nucleic acid using 1 U of Benzonase (Sigma) and 0.5% Tween 20 (Sigma) and incubated at 37°C for 5 min. Subsequently, the DNA nucleic acid was extracted using a QIAamp UCP pathogen minikit. Qubit 4.0 (Thermo Fisher Scientific, MA, USA) was used to measure extracted DNA concentrations, which were used to construct metagenomics libraries. The inspected and qualified library was sequenced on the Nextseq 550 platform (Illumina, San Diego, USA). For targeted NGS, the constructed library from each sample was used for hybrid capture-based enrichment of microbial probes. To remove adapter and low-quality, low complexity, and short reads of < 35 bp, raw data generated by the sequencing were filtered. The clean reads were blasted against a microbial database downloaded from the National Center for Biotechnology Information. Finally, microbial information at the species level can be obtained.

## Results

### Hospitalization and infections in patients with COPD

Recurrent acute exacerbations and hospitalization are crucial characteristics of severe COPD. We observed 56 patients with COPD admitted to the hospital from July 2020 to January 2024, most of whom were hospitalized with a combination of lung infections. During hospitalization, 94.6% of patients received antibiotics, 21.4% underwent mechanical ventilation, and 64.2% faced hospitalization costs over 20,000 RMB. Additionally, 51.8% were hospitalized multiple times during follow-up, with a mortality rate of 5.4% (**Supplementary Table 2**). The mNGS results suggested that *Pseudomonas aeruginosa* and Human gammaherpesvirus 4 were the most common bacterial and viral pathogens (**Supplementary Figure 1**).

### Instruments for druggable genes

We used eQTLs from whole blood and lung tissues to intersect with druggable genes to obtain druggable eQTLs. Finally, cis-eQTLs for 2529 druggable genes from eQTLGen’s whole blood and 1042 druggable genes from GTEx v.8’s lung tissues were extracted, both of which were used for SMR analyses with COPD and lung function as outcomes.

Among the genes identified by SMR/HEIDI and colocalization analysis, we obtained cis-pQTL data for 9 genes from the proteomics summary data of whole blood samples provided by Ferkingstad et al.

Our exposure and outcome samples were both predominantly European, so it can be assumed that the genetic variants used as instrumental variables (cis-eQTLs and cis-pQTLs) had a consistent role between the two different sample sets. The exposure and outcome samples were from completely independent studies, so there was no participant overlap.

### Druggable targets discovered by eQTL SMR and HEIDI test

We performed SMR analysis using doctor-diagnosed COPD GWAS summary data from the FinnGen consortium. Initially, we identified 3 genes (SLC22A5, C2, and GPC2) whose expression levels in whole blood were found to be correlated with doctor-diagnosed COPD (corrected for *P*-value by Bonferroni, *P*_HEIDI≥0.01, see **Supplementary Table 3** for detailed data).

We utilized the FEV1/FVC<0.7 GWAS summary data from the UK biobank, and identified 23 genes whose expression levels in whole blood or lung tissues were associated with spirometry-defined COPD (corrected for *P*-value by Bonferroni, *P*_HEIDI≥0.01, see **Supplementary Table 3** for detailed data).

The SMR analysis using FEV1 GWAS summary data identified 36 genes that were significantly associated with FEV1. Out of these genes, six were also found to be associated with spirometry-defined COPD.

### Druggable genes proposed after colocalization

**Figure 3:**
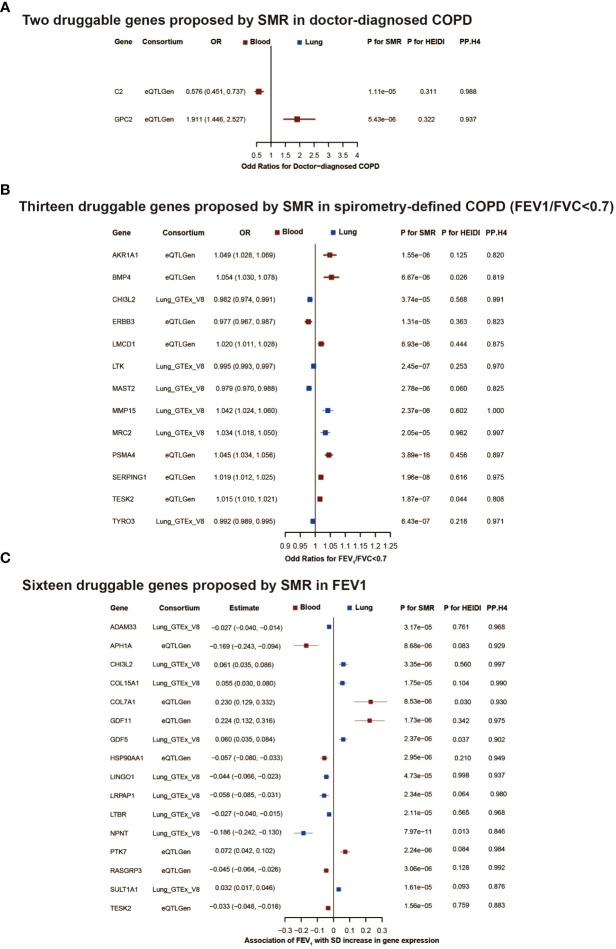
The druggable genes proposed by summary-data-based Mendelian randomization in COPD or lung function. The forest plot showed changes in doctor-diagnosed COPD risk, spirometry-defined COPD (FEV1/FVC<0.7) risk, and FEV1 level with an increase in gene expression per standard deviation. All genes passed eqtl SMR, HEIDI test, and colocalization analysis. The colors denote different tissues (red: blood, blue: brain tissue). In **(A, B)**, an OR<1 indicates a decrease in risk, while an OR>1 means an increase in risk. In **(C)**, a negative estimate corresponds to a decrease in FEV1, whereas a positive estimate denotes an increase in FEV1. COPD, chronic obstructive pulmonary disease; FEV1, forced expiratory volume in 1 second; FVC, forced vital capacity; OR, odd ratios; SD, standard deviation; SMR, summary-data-based mendelian randomization.

Furthermore, colocalization analysis identified 2, 13, and 16 genes that shared the same SNPs (PP.H4 > 0.8 and/or PH4/PH3 > 2, **Supplementary Table 4**) in the results of doctor diagnosed COPD, FEV1/FVC<0.7 and FEV1, respectively (**Figure 3**).

### Druggable targets supported by eQTL two-sample MR

We performed eQTL two-sample MR (**Table 1**; **Supplementary Table 5**) on druggable genes that had passed the colocalization analysis to assess the consistency of direction and statistical significance.

**Table 1:** Multi-evidences supporting druggable genes whose expression was significantly associated with COPD and lung function.

Gene	Tissue	SMR with HEIDI test		Coloc analysis	Protein SMR with HEIDI test		Two-sample MR		Consortium	High priority
		Support	Direction	Support	Support	Direction	Support	Direction		
Druggable genes whose expression was significantly associated with doctor-diagnosed COPD										
C2	Whole-blood	√	–	√	x	–	x	+	eQTLgen	x
GPC2	Whole-blood	√	+	√			√	+	eQTLgen	√
Druggable genes whose expression was significantly associated with spirometry-defined COPD (FEV1/FVC<0.7)										
TESK2	Whole-blood	√	+	√			√	+	eQTLGen	√
AKR1A1	Whole-blood	√	+	√			√	+	eQTLGen	√
LMCD1	Whole-blood	√	+	√			√	+	eQTLGen	√
SERPING1	Whole-blood	√	+	√	x	–	√	+	eQTLGen	x
ERBB3	Whole-blood	√	–	√	√	–	√	–	eQTLGen	√
PSMA4	Whole-blood	√	+	√			√	+	eQTLGen	√
MAST2	Lung	√	–	√			√	–	GTEx_V8	√
CHI3L2	Lung	√	–	√			x		GTEx_V8	x
MMP15	Lung	√	+	√					GTEx_V8	√
MRC2	Lung	√	+	√			√	+	GTEx_V8	√
BMP4	Whole-blood	√	+	√			√	+	eQTLGen	√
LTK	Lung	√	–	√			√	–	GTEx_V8	√
TYRO3	Lung	√	–	√	x	+	√	–	GTEx_V8	x
Druggable genes whose expression was significantly associated with FEV1										
APH1A	Whole-blood	√	–	√			√	–	eQTLgen	√
CHI3L2	Lung	√	+	√			√	+	GTEx.v8	√
COL7A1	Whole-blood	√	+	√			x	+	eQTLgen	x
GDF11	Whole-blood	√	+	√			x	–	eQTLgen	x
GDF5	Lung	√	–	√			x	+	GTEx.v8	x
PTK7	Whole-blood	√	+	√	√	–	√	+	eQTLgen	√
RASGRP3	Whole-blood	√	–	√			√	–	eQTLgen	√
HSP90AA1	Whole-blood	√	–	√			√	–	eQTLgen	√
TESK2	Whole-blood	√	–	√			√	–	eQTLgen	√
ADAM33	Lung	√	–	√			√	–	GTEx.v8	√
COL15A1	Lung	√	+	√	√	+	√	+	GTEx.v8	√
LINGO1	Lung	√	–	√	√	–	√	–	GTEx.v8	√
LRPAP1	Lung	√	–	√	x	–	√	–	GTEx.v8	X
LTBR	Lung	√	–	√			√	–	GTEx.v8	√
NPNT	Lung	√	–	√	x	+	√	–	GTEx.v8	X
SULT1A1	Lung	√	+	√			√	+	GTEx.v8	√

COPD, chronic obstructive pulmonary disease; FEV1, forced expiratory volume in 1 second; FVC, forced vital capacity; √, pass; x, fail; +, positive; -, negative; blank not possible to test; SMR, summary-data-based mendelian randomization; HEIDI test, heterogeneity in dependent instruments; Coloc, colocalization; MR, Mendelian randomization; High priority means there are no contradictory analysis results.

### Druggable targets supported by protein SMR

We further performed protein SMR analysis (**Table 1**; **Supplementary Table 5**). Of the identified genes, 1 (GPC2) was associated with doctor-diagnosed COPD, 10 (TESK2, AKR1A1, LMCD1, ERBB3, PSMA4, MAST2, MMP15, MRC2, BMP4, and LTK) with FEV1/FVC <0.7, and 11 (APH1A, CHI3L2, PTK7, RASGRP3, HSP90AA1, TESK2, ADAM33, COL15A1, LINGO1, LTBR, and SULT1A1) with FEV1 in protein SMR results, without contradicting the expression SMR results. These genes were categorized as high priority.

In addition, we tested the underlying assumptions and sensitivity analyses for two-sample MR (**Supplementary Table 6**). The F-Statistic for the gene instrumental variables were all greater than 10. The Egger test indicated no evidence of horizontal pleiotropy for 16 genes and suggested that the IVW results could be used as the primary ones. The Steiger test confirmed the correct causal direction.

### Repurposing opportunities or adverse effects for existing drugs

Among the high-priority drug-targeted genes, MMP15 is associated with Montelukast and COL-3 (NSC-683551), while PSMA4 is linked to MARIZOMIB. These three drugs are small molecule inhibitors, which may reduce the risk of spirometry-defined COPD. On the other hand, the APH1A gene, which codes for the gamma-secretase subunit APH-1A, appears to negatively impact FEV1 (β<0 in MR analysis). Several existing inhibitors against this target (e.g., TARENFLURBIL, BEGACESTAT, SEMAGACESTAT, NIROGACESTAT, RG-4733, and AVAGACESTAT) may counteract this harmful effect. Additionally, high expression of the ERBB3 gene may lower the risk of spirometry-defined COPD. However, various existing antitumor drugs, which are inhibitors of this target, may increase the risk of spirometry-defined COPD (**Table 2**).

**Table 2:** Repurposing opportunities or adverse effects for existing drugs on COPD and lung function.

Gene	Expression in tissue	Effect of gene	Outcome	Gene coding protein	Compound (drug) name	Action type	Molecule type	Current indication	Clinical phase	Evidence from
Repurposing opportunities for existing drugs										
PSMA4	Whole-blood	Increase risk	FEV1/FVC<0.7	Proteasome subunit alpha type-4	MARIZOMIB	Inhibitor	Small molecule	Cancer	Phase 3	ChEMBL
MMP15	Lung	Increase risk	FEV1/FVC<0.7	Matrix metalloproteinase-15	COL-3 (NSC-683551)	Inhibitor	Single protein	Cancer	Phase 1	Clinicaltrials.gov
					Montelukast	Inhibitor	Small molecule	Asthma	Approved	Clinicaltrials.gov
APH1A	Whole-blood	Impair	FEV1	Gamma-secretase subunit APH-1A	TARENFLURBIL	Inhibitor	Small molecule	Alzheimer Disease, Dementia	Phase 3	ChEMBL
					BEGACESTAT	Inhibitor	Small molecule	Alzheimer Disease	Phase 1	ChEMBL
					SEMAGACESTAT	Inhibitor	Small molecule	Alzheimer Disease	Phase 3	ChEMBL
					NIROGACESTAT	Inhibitor	Small molecule	Cancer	Phase 3	ChEMBL
					RG-4733	Inhibitor	Small molecule	Cancer	Phase 2	ChEMBL
					AVAGACESTAT	Inhibitor	Small molecule	Alzheimer Disease	Phase 2	ChEMBL
LTBR	Lung	Impair	FEV1	Tumor necrosis factor receptor superfamily member 3	HCBE-11	Inhibitor	Antibody	Cancer	Phase 1	ChEMBL
Adverse effects of existing drugs										
ERBB3	Whole-blood	Decrease risk	FEV1/FVC<0.7	Receptor tyrosine-protein kinase erbB-3	CDX-3379	Inhibitor	Antibody	Cancer	Phase 2	ChEMBL
					MM-121	Antagonist	Antibody	NSCLC and other cancer	Phase 2	ChEMBL
					ELGEMTUMAB	Inhibitor	Antibody	Cancer	Phase 1	ChEMBL
					DULIGOTUZUMAB	Inhibitor	Antibody	Cancer	Phase 2	ChEMBL
					SAPITINIB	Inhibitor	Small molecule	NSCLC and other cancer	Phase 2	ChEMBL
					LUMRETUZUMAB	Inhibitor	Antibody	NSCLC and other cancer	Phase 1	ChEMBL
					AMG-888	Inhibitor	Antibody	NSCLC and other cancer	Phase 2	ChEMBL
					SERIBANTUMAB	Inhibitor	Antibody	Cancer	Phase 2	ChEMBL
					AV-203	Inhibitor	Antibody	Cancer	Phase 1	ChEMBL
					PATRITUMAB- DERUXTECAN	Binding-Agent	Antibody	NSCLC and other cancer	Phase 3	ChEMBL
					PATRITUMAB	Inhibitor	Antibody	NSCLC and other cancer	Phase 3	ChEMBL
					ISTIRATUMAB	Inhibitor	Antibody	Cancer	Phase 2	ChEMBL
COL15A1	Lung	improve	FEV1		OCRIPLASMIN	Inhibitor	Enzyme	Cancer	Approved	ChEMBL
					COLLAGENASE CLOSTRIDIUM HISTOLYTICUM	Inhibitor	Enzyme	Cancer	Approved	ChEMBL

Repurposing opportunities or adverse effect data from druggable gene list, ClinicalTrials (https://www.ClinicalTrials.gov), or ChEMBL release.

COPD, chronic obstructive pulmonary disease; FEV1, forced expiratory volume in 1 second; FVC, forced vital capacity.

### Phenome-wide MR evaluated side-effects of druggable genes

**Figure 4:**
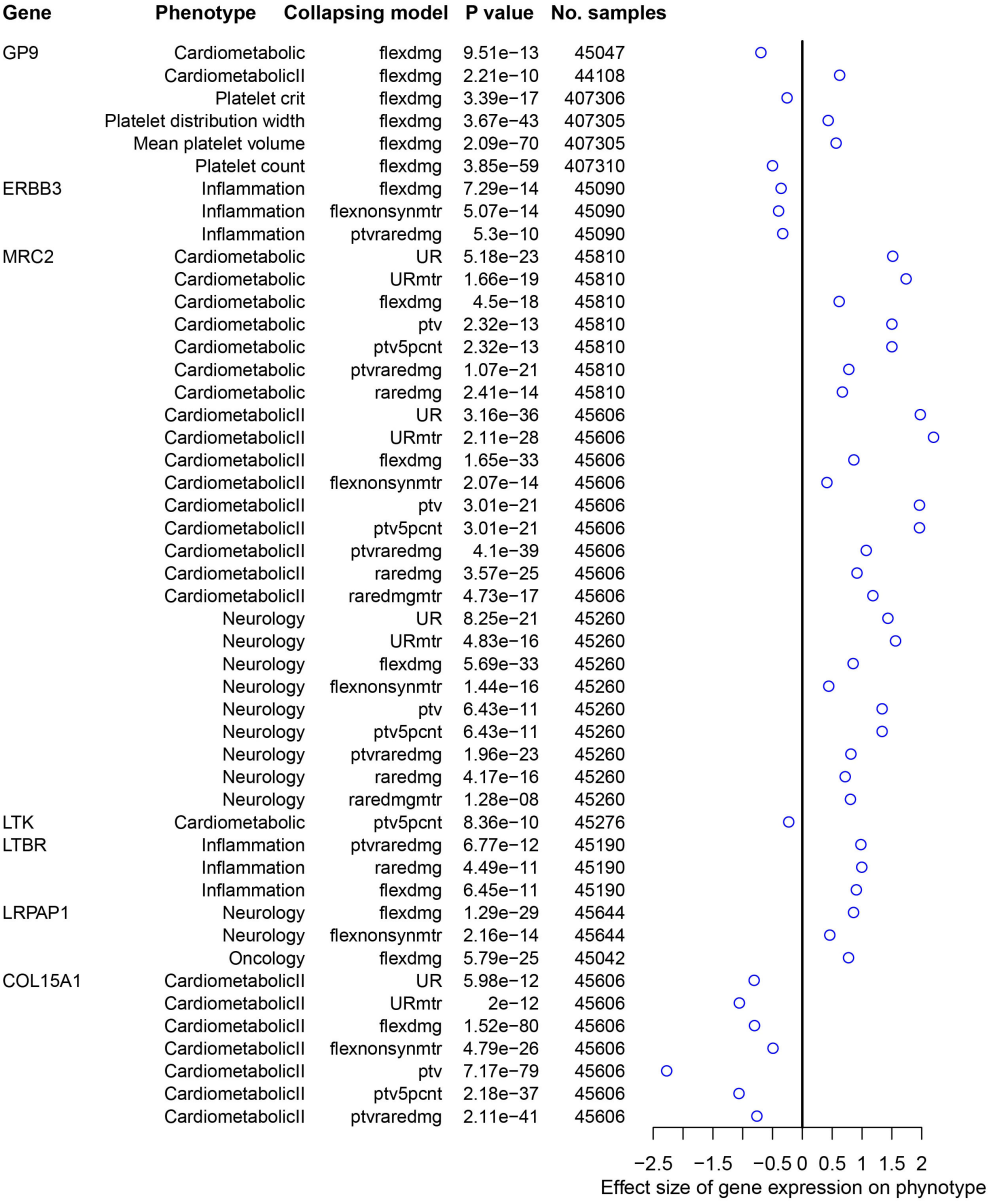
Traits significantly associated with druggable genes using AstraZeneca PheWAS portal. The effect size represents the extent of the impact of genetic variation on the phenotype, with a positive value indicating a positive correlation and a negative value indicating a negative correlation. PheWAS, phenome-wide association study.

The PheWAS analysis revealed that increased GP9 expression may have adverse effects on platelets in addition to possibly increasing the risk of spirometry-defined COPD. Conversely, increased ERBB3 expression is associated with a reduced risk of spirometry-defined COPD and inflammation suppression. Moreover, increased MRC2 expression affects cardio metabolism and the nervous system (**Figure 4**).

## Discussion

The real-world observational study emphasized the urgency and necessity of developing new drugs for COPD. In this study, we aimed to uncover druggable genes and explore drug repurposing possibilities for COPD and lung function. Through a comprehensive multi-omics approach, which encompassed SMR analysis with eQTL and pQTL data, colocalization analyses, and two-sample MR (MR Egger, Weighted Median, and Inverse Variance Weighted), we successfully identified and validated 22 potential druggable genes. Among these genes, MMP15, APH1A, LTBR, and PSMA4 emerged as potential targets for existing drugs. The potential effects of these targets were also investigated using PheWAS analysis. To our knowledge, this is the first study to use MR methods to identify drug targets for COPD and lung function, utilizing the largest publicly available QTL data and COPD GWAS data to date.

Montelukast and COL-3 (NSC-683551) have been identified as inhibitors of MMP expression, including MMP15, which plays a crucial role in extracellular matrix breakdown relevant to lung function. Previous studies have revealed the association of the MMP15 gene with lung function and its specific expression in multiple lung cell types (Soler Artigas et al., 2011; Chun et al., 2022). Moreover, several studies have linked various MMP family members, including MMP-9, MMP-12, and MMP-15, to COPD through the degradation of the alveolar extracellular matrix (Babusyte et al., 2007; Gharib et al., 2018; Wells et al., 2018; Zhou et al., 2020). Our findings suggest that inhibiting MMP15 could be an effective strategy to prevent spirometry-defined COPD. Montelukast, a common inhibitor of allergic reactions, has been widely prescribed for the treatment of asthma and chronic cough (von Mutius and Drazen, 2010; Wang et al., 2014). Since controlling asthma and airway hyperresponsiveness can help prevent spirometry-defined COPD (Couillard et al., 2023), it’s likely that Montelukast could reduce the risk of spirometry-defined COPD by decreasing MMP levels in patients. On the other hand, COL-3, despite its broad inhibitory effects on MMPs, is limited in clinical use due to side effects like photosensitization. This highlights the need for maintaining a balance between efficacy and safety when considering strategies for lung function protection.

MARIZOMIB is a small molecule inhibitor that targets PSMA4. Our results suggest that PSMA4 expression increases the risk of spirometry-defined COPD. The significant association between PSMA and severe COPD has been confirmed by a genome-wide association study, which underscored the crucial role of PSMA4 in both whole blood and lung tissue (Sakornsakolpat et al., 2018). Additionally, extensive research investigating genetic variants on chromosome 15q25.1 in relation to COPD has consistently identified PSMA4 as a gene exhibiting substantial associations with methylation differences in this genomic region (Nedeljkovic et al., 2018). Such findings are in alignment with our results. However, the potential clinical application of PSMA4 inhibitors, which are currently used in oncology, for COPD treatment remains to be explored.

Our analysis revealed a negative causal association between increased levels of ERBB3 expression in blood, indicating that the expression of this gene could act as a protective factor against spirometry-defined COPD. Although studies on the association of the ERBB3 gene with COPD are limited, one study has shown that mRNA levels of ERBB4 increase progressively from non-smokers to non-COPD smokers and then to COPD patients and is positively correlated with airflow obstruction severity (Anagnostis et al., 2013). This suggests that the ERBB receptors may contribute to the development or progression of COPD. However, an increase in ERBB3 expression does not necessarily indicate that the gene promotes the development of COPD. Instead, it may be a protective response of the organism to control the level of inflammation. Phenome-wide MR analysis in this study suggests that increased ERBB3 expression is associated with the suppression of inflammation. Evidence has shown that ERBB signaling can inhibit the production of TNF-α induced by LPS in immune activation associated with chronic systolic heart failure (Ryzhov et al., 2017). Therefore, the relationship between ERBB3 and COPD, as well as its mechanism of action, such as its inhibition of inflammation, requires further verification.

An association between increased levels of LMCD1 expression in the blood and an increased risk of spirometry-defined COPD was revealed in this study. It has been shown that LMCD1 plays an important role in the development of lung fibrosis and affects the properties of lung myofibroblasts (Jiang et al., 2021). In particular, in cases of systemic sclerosis-associated lung fibrosis, LMCD1 interacts with serum response factors in lung fibroblasts, which leads to increased contractile activity of lung myofibroblasts. This suggests that LMCD1 is a pro-fibrotic molecule that contributes to myofibroblast activation and sustained fibrotic proliferation (Bogatkevich et al., 2023). Lung fibrosis and lung myofibroblasts also play important roles in the pathophysiology of COPD, and it is reasonable to hypothesize that LMCD1 could potentially contribute to lung tissue fibrosis, thereby increasing the risk of spirometry-defined COPD.

Our study has also discovered several previously unreported genes associated with COPD or lung function. Specifically, we found that increased expression of APH1A and RASGRP3 is likely to lead to decreased FEV1, while increased expression of CHI3L2 is associated with increased FEV1. Besides, heightened expression of GPC2 is linked to an increased risk of COPD, while increased expression of TESK2, AKR1A1, and MRC2 may result in an increased risk of spirometry-defined COPD. Increased expression of MAST2 appears to lower the risk of spirometry-defined COPD. Although no previous reports have been found on the direct association of these genes with COPD or lung function, they have well-defined roles in various other conditions, such as tumors, Alzheimer’s disease, and inflammation. For example, GPC2 has been identified as a prognostic marker for several types of tumors (Liu et al., 2021; Chen et al., 2022), PTK7 shows potential as a target for CAR T-cell therapies in lung cancer treatment (Ma et al., 2023), APH1A (Todd et al., 2022) is involved in the development of Alzheimer’s disease, and RasGRP3 acts to limit the inflammatory response during low-intensity pathogen infections (Lee et al., 2023). While our findings provide new insights into the potential involvement of these genes in COPD development, further studies are needed to determine their exact role in the disease.

Our study has several limitations. Firstly, while MR offers insights into causality, it presumes a linear relationship between exposure and outcome, which may not capture non-linear or U-shaped exposure-response relationships. Secondly, the QTLs used in our study may only show small differences in gene expression levels, which might fail to fully capture the gene’s potential effects. Furthermore, the QTLs and FEV1 data included some individuals of non-European ancestry, whereas the Doctor-diagnosed COPD and FEV1/FVC<0.7 GWAS populations consisted of Europeans only. These differences in population may introduce potential bias in MR effect estimates due to differences in genetic background and chain imbalance patterns. Finally, this study mainly focused on European populations, which limits the generalizability of the findings to other ethnic groups. Therefore, further research and validation are necessary to generalize the results to other ethnicities.

Our study has identified potential therapeutic targets for COPD. In the future, well-established drugs like Montelukast, which targets the MMP15 gene, and Marizomib, targeting the PSMA4 gene, could be prioritized for clinical trials. However, the disease-modifying potential of many druggable genes requires further experimental validation.

## Conclusion

This study found and validated 22 potential druggable genes that show promise for COPD and lung function. Our findings provide genetic evidence supporting the potential therapeutic benefits of targeting these genes in the treatment of COPD. Clinical trials prioritizing existing drugs and novel medications targeting these identified druggable genes could potentially increase the likelihood of successful treatments.

## Data availability statement

The datasets presented in this study can be found in online repositories. The names of the repository/repositories and accession number(s) can be found in the article/**Supplementary Material**.

## Author contributions

ZW: Conceptualization, Data curation, Writing – original draft, Writing – review & editing, Formal analysis, Investigation, Methodology, Project administration, Resources, Software, Supervision, Validation, Visualization. SL: Conceptualization, Data curation, Writing – original draft, Writing – review & editing, Formal analysis, Investigation, Methodology, Project administration, Resources, Software, Supervision, Validation, Visualization. GC: Formal analysis, Writing – original draft, Writing – review & editing. YG: Formal analysis, Writing – original draft, Writing – review & editing. HY: Data curation, Writing – original draft, Writing – review & editing. YL: Data curation, Writing – original draft, Writing – review & editing. JL: Formal analysis, Writing – original draft, Writing – review & editing. SZ: Formal analysis, Writing – original draft, Writing – review & editing. JH: Formal analysis, Writing – original draft, Writing – review & editing, Funding acquisition. JZ: Conceptualization, Funding acquisition, Writing – original draft, Writing – review & editing, Resources, Supervision.

## Supplementary material

Supplementary Figure 1 Pathogen profiles of COPD patients with hospitalization.
