# Supplementary figures and images for: Mendelian randomization analysis identifies druggable genes and drugs repurposing for chronic obstructive pulmonary disease
Source: Front Cell Infect Microbiol. 2024 Apr 10;14:1386506. doi: 10.3389/fcimb.2024.1386506 (PMC11039854; doi:10.3389/fcimb.2024.1386506)

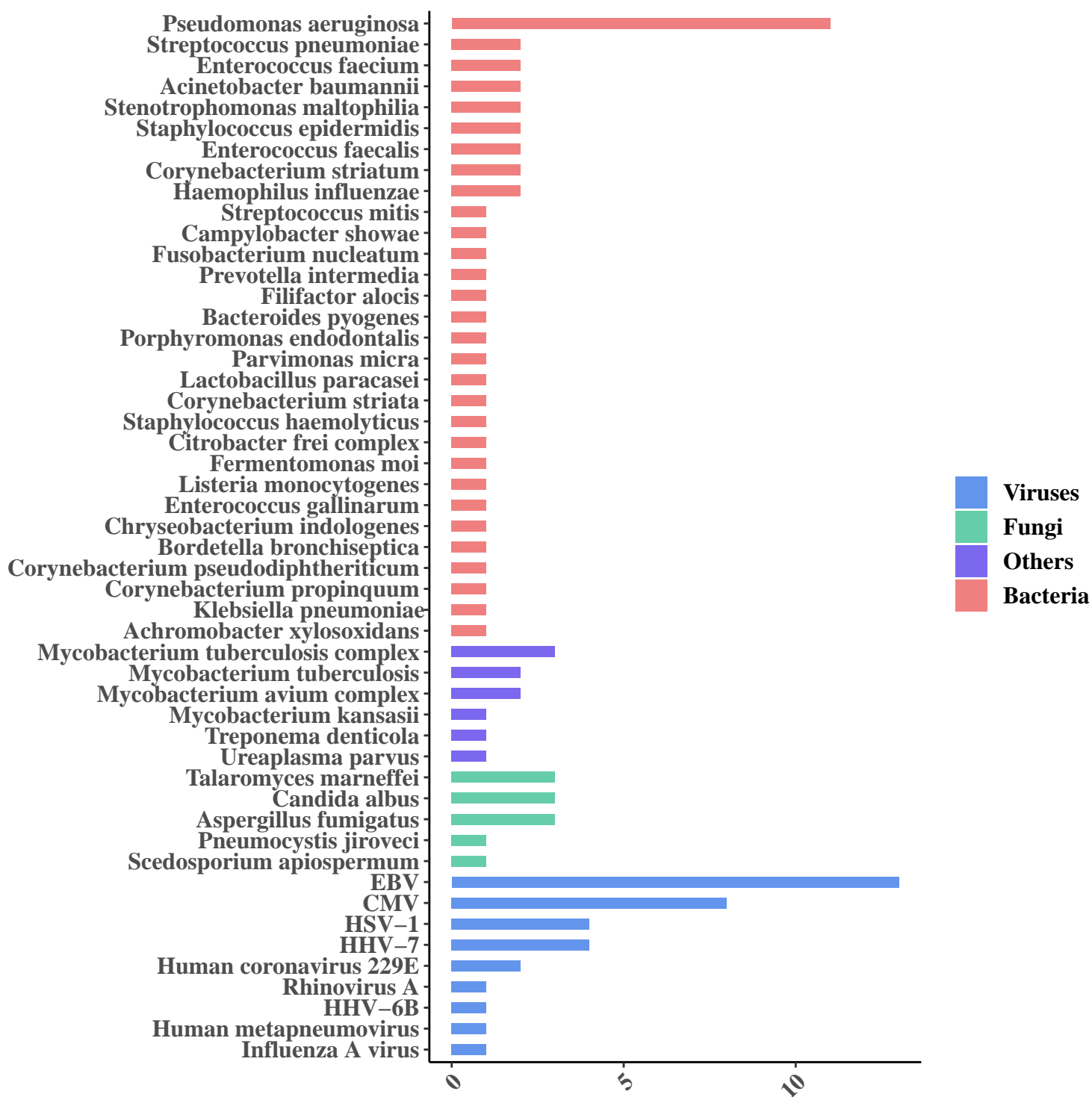

Supplement: Supplementary Figure 1 — Pathogen profiles of COPD patients with hospitalization. [file Image_1.pdf]
