# Supplementary material for: Mendelian randomization analysis identifies druggable genes and drugs repurposing for chronic obstructive pulmonary disease
Source: Front Cell Infect Microbiol. 2024 Apr 10;14:1386506. doi: 10.3389/fcimb.2024.1386506 (PMC11039854; doi:10.3389/fcimb.2024.1386506)
Supplement: Supplementary file 2 [file Table_1.docx]

**Supplementary Table 1.** Information of QTL and GWAS datasets.

| **Type of dataset** | **Data subtype** | **Resource** | **Sample size** | **Population** | **Reference** | **Download Site** |
| --- | --- | --- | --- | --- | --- | --- |
| **QTL** | **cis-eQTL** | eQTLGen Consortium (Whole-blood) | 25,482 | Predominantly European | PMID: 34475573 | www.eqtlgen.org |
|  |  | GTEx v.8 Consortium  (Lung) | 515 | Predominantly European | PMID: 32913098 | www.gtexportal.org |
|  | **cis-pQTL** | Egil Ferkingstad et al. pQTL summary data  (Whole-blood) | 35,559 | Predominantly European | PMID: 28240269 | www.decode.com/summarydata |
| **GWAS summary** | **Doctor diagnosis COPD** | FinnGen consortium | case: 16,410  control: 283,589 | European | NA | www.finngen.fi/en/access_results |
|  | **FEV1** | UK Biobank and SpiroMeta | 321,047 | Predominantly European | PMID: 30804560 | gwas.mrcieu.ac.uk/datasets/ebi-a-GCST007432 |
|  | **Spirometry defined COPD (FEV1/FVC<0.7)** | UK Biobank | case: 55,907  control: 297,408 | European | PMID: 33574079 | gwas.mrcieu.ac.uk/datasets/ieu-b-106/ |
