# Supplementary material for: Mendelian randomization analysis identifies druggable genes and drugs repurposing for chronic obstructive pulmonary disease
Source: Front Cell Infect Microbiol. 2024 Apr 10;14:1386506. doi: 10.3389/fcimb.2024.1386506 (PMC11039854; doi:10.3389/fcimb.2024.1386506)
Supplement: Supplementary file 3 [file Table_2.docx]

eTable 2 Outcomes of COPD patients*

| Patients | Sex | Age | Best FEV1%pred at hospitalization** | Years of follow-up | Total number of admissions | Average total cost per admission | Use of antibiotics | Use of mechanical ventilation | Deaths during hospitalization |
| --- | --- | --- | --- | --- | --- | --- | --- | --- | --- |
| P1 | Male | 56 | 45 | 1 | 1 | 15875 | Yes | No | No |
| P2 | Male | 65 | NA | 1 | 2 | 77442 | Yes | No | No |
| P3 | Male | 82 | NA | 1 | 1 | 107625 | Yes | Yes | No |
| P4 | Male | 68 | 43 | 1 | 3 | 219922 | Yes | Yes | Yes |
| P5 | Male | 59 | NA | 1 | 2 | 22951 | Yes | No | No |
| P6 | Male | 76 | 50 | 4 | 7 | 13628 | Yes | No | No |
| P7 | Male | 85 | 92 | 2 | 3 | 20785 | Yes | No | No |
| P8 | Male | 72 | NA | 1 | 2 | 2741354 | Yes | Yes | Yes |
| P9 | Male | 62 | 69 | 1 | 1 | 16783 | Yes | No | No |
| P10 | Male | 75 | NA | 1 | 4 | 26591 | Yes | No | No |
| P11 | Male | 84 | NA | 1 | 1 | 14705 | Yes | No | No |
| P12 | Male | 73 | 84 | 1 | 1 | 15840 | Yes | No | No |
| P13 | Female | 63 | 48 | 4 | 7 | 21416 | Yes | No | No |
| P14 | Male | 58 | 71 | 1 | 2 | 14632 | Yes | No | No |
| P15 | Male | 87 | NA | 2 | 3 | 99977 | Yes | Yes | Yes |
| P16 | Female | 59 | 59 | 2 | 2 | 18381 | Yes | No | No |
| P17 | Female | 79 | 46 | 1 | 1 | 23827 | No | No | No |
| P18 | Male | 70 | NA | 1 | 1 | 32784 | Yes | Yes | No |
| P19 | Male | 77 | NA | 3 | 3 | 84301 | Yes | Yes | No |
| P20 | Male | 56 | 52 | 1 | 1 | 18065 | Yes | No | No |
| P21 | Male | 65 | 36 | 3 | 3 | 37039 | Yes | No | No |
| P22 | Male | 69 | NA | 1 | 1 | 407937 | Yes | Yes | No |
| P23 | Male | 72 | 31 | 2 | 19 | 15027 | Yes | No | No |
| P24 | Male | 69 | NA | 1 | 5 | 16541 | Yes | No | No |
| P25 | Male | 63 | 32 | 1 | 3 | 16739 | Yes | No | No |
| P26 | Male | 85 | 37 | 1 | 1 | 78201 | Yes | Yes | No |
| P27 | Male | 66 | 56 | 3 | 6 | 32911 | Yes | No | No |
| P28 | Male | 49 | 39 | 2 | 4 | 20158 | Yes | No | No |
| P29 | Male | 71 | 30 | 2 | 6 | 38827 | Yes | Yes | No |
| P30 | Male | 53 | NA | 1 | 1 | 33615 | Yes | No | No |
| P31 | Male | 74 | 104 | 1 | 1 | 37178 | Yes | No | No |
| P32 | Male | 65 | 65 | 3 | 20 | 10050 | Yes | No | No |
| P33 | Female | 71 | 46 | 2 | 2 | 22207 | Yes | No | No |
| P34 | Female | 61 | 82 | 1 | 1 | 18502 | Yes | No | No |
| P35 | Male | 69 | 25 | 2 | 3 | 7026 | Yes | No | No |
| P36 | Male | 68 | 76 | 2 | 4 | 40592 | Yes | No | No |
| P37 | Male | 74 | NA | 2 | 9 | 18043 | No | No | No |
| P38 | Female | 66 | 36 | 1 | 1 | 29881 | Yes | Yes | No |
| P39 | Male | 77 | NA | 1 | 3 | 19241 | No | No | No |
| P40 | Male | 55 | 12 | 1 | 1 | 24903 | Yes | No | No |
| P41 | Female | 64 | 57 | 1 | 1 | 15820 | Yes | No | No |
| P42 | Male | 54 | NA | 1 | 1 | 8796 | Yes | No | No |
| P43 | Male | 49 | 39 | 1 | 1 | 19947 | Yes | No | No |
| P44 | Male | 83 | NA | 1 | 2 | 26591 | Yes | Yes | No |
| P45 | Female | 85 | 59 | 1 | 1 | 20325 | Yes | No | No |
| P46 | Male | 74 | 88 | 1 | 1 | 27220 | Yes | No | No |
| P47 | Male | 91 | 33 | 1 | 1 | 33371 | Yes | No | No |
| P48 | Male | 62 | 33 | 1 | 1 | 18525 | Yes | No | No |
| P49 | Male | 74 | 96 | 1 | 5 | 18002 | Yes | No | No |
| P50 | Female | 76 | NA | 1 | 1 | 25757 | Yes | No | No |
| P51 | Male | 69 | 32 | 1 | 1 | 15825 | Yes | No | No |
| P52 | Male | 74 | 20 | 1 | 2 | 12855 | Yes | No | No |
| P53 | Male | 65 | 64 | 1 | 1 | 21013 | Yes | No | No |
| P54 | Male | 62 | 33 | 1 | 1 | 25059 | Yes | No | No |
| P55 | Male | 56 | 56 | 1 | 3 | 15844 | Yes | Yes | No |
| P56 | Male | 76 | 55 | 1 | 1 | 30036 | Yes | No | No |

*Diagnosis of COPD at discharge

**Pulmonary function tests was not performed during hospitalisation
